# Association between polymorphisms in the estrogen receptor alpha gene and osteoarthritis susceptibility: a meta-analysis

**DOI:** 10.1186/s12891-015-0506-5

**Published:** 2015-02-27

**Authors:** Yan Ren, Bo Tan, Peijing Yan, Yi You, Yanqiao Wu, Yue Wang

**Affiliations:** Department of Epidemiology and Biostatistics, West China School of Public Health, Sichuan University, Chengdu, 610041 People’s Republic of China; Department of Orthopedics, Sichuan Academy of Medical Sciences, Sichuan Provincial People’s Hospital, Chengdu, 610072 People’s Republic of China; Department of Preventive Health Care, The People’s Hospital of Dazu District, Chongqing, 402360 People’s Republic of China; Department of Medical Record Management, West China Second University Hospital, Sichuan University, Chengdu, 610041 People’s Republic of China

**Keywords:** Estrogen receptor, Osteoarthritis, Polymorphism, Meta-analysis

## Abstract

**Background:**

Osteoarthritis (OA) is a common chronic disease of the joints. Genetic factors may play a role in its development, and polymorphisms in the estrogen receptor alpha gene (*ERα*) have been associated with OA. However, previous studies into this relationship have reported inconsistent results, so we aimed to systematically review the association between *ERα* polymorphisms and OA susceptibility.

**Methods:**

We conducted a comprehensive literature search of Ovid MEDLINE, EMBASE, CBM, and PubMed databases, and Google scholar, and identified 11 eligible studies that examined the association between *ERα* polymorphisms and OA susceptibility. We carried out a meta-analysis of these studies based on *ERα Xba*I (rs9340799) and *Pvu*II (rs2234693) genotypes.

**Results:**

Seventeen comparisons involving 10 European and seven Asian populations of 5,325 OA patients and 10,834 controls were included in the study. The *ERα Xba*I polymorphism were significantly associated with OA in Europeans (AA vs. AG + GG: OR = 1.17, 95% confidence interval (CI) = 1.02–1.34, *P* = 0.03; AG vs. AA + GG: OR = 0.86, 95% CI = 0.75–0.99, *P* = 0.04) but not in Asian populations. No association was found between OA and the *ERα Pvu*II polymorphism in any population (C vs. T, OR = 0.98, 95% CI = 0.93–1.03, *P* = 0.37; CC vs. TT + CT, OR = 0.97, 95% CI = 0.89–1.06, *P* = 0.55; CT vs. CC + TT, OR = 0.99, 95% CI = 0.92–1.06, *P* = 0.75; TT vs. CC + CT, OR = 1.01, 95% CI =0.92–1.12, *P* = 0.79).

**Conclusions:**

This study suggested that there may be a weak relationship between the *ERα Xba*I polymorphism and OA in Europeans but not Asians, and that the *ERα Pvu*II polymorphism was not associated with OA in either population. However, large well-designed studies are necessary to confirm these results in more homogeneous populations.

## Background

Osteoarthritis (OA) is the most common joint disease worldwide, and primarily affects the knees, hips, hands, and spine. It is a leading cause of disability among older individuals and also affects their quality of life [1]. It is characterized by the progressive degeneration of articular cartilage, and by subchondral sclerosis resulting in pain and joint stiffness [2].

The etiology of OA is multifactorial, including genetic and environmental risk factors. Associated genes include *GDF5* [3], *ASPN* [4], *FRZB* [5], and *COL2A1* [6], while environmental factors may include obesity [7-9], history of knee injury [10], occupational activities [11,12], sex hormones and structural changes [13], meniscectomy [14], gender, and age [15]. Twin-pair and family genetic data show that more than 50% of OA can be attributed to genetic factors [16]. A gender difference is also apparent, with females having a greater prevalence of OA after the age of 50 years [17]. Additionally, the disease is more common among European populations [18]. The observation that the estrogen receptor (ER) is expressed in human articular chondrocytes and bone cells suggests that it may be involved in the etiology of OA [19].

The ER has two isoforms: ERα and ERβ. *ERα* expression affects the growth of bone cells, while *ERβ* participates in the formation and resorption of bone [20]. *ERα* is located on chromosome 6q25.1 and contains eight exons and seven introns [21], as well as two common restriction fragment length polymorphisms (RFLPs): *Xba*I and *Pvu*II. The *Xba*I RFLP detects an A–G substitution at position 351 (−351int A/G; rs9340799), while *Pvu*II detects a T–C substitution at position 397 (−397int T/C; rs2234693). A previous meta-analysis confirmed the association between bone mineral density and *ERα* [22].

A number of studies have investigated the association between *ERα* polymorphisms and the risk of OA in different populations, but the results are inconsistent. Some discovered that *ERα* polymorphisms were associated with an increased risk of OA [23-28], while others found no association with OA risk [28,29], or an association with a reduced risk of OA [30-34]. To our knowledge, no systematic review has examined the evidence for a relationship between *ERα* polymorphisms and OA. Therefore, we conducted a meta-analysis to analyze the association between *ERα* polymorphisms and OA susceptibility.

## Methods

This systematic review was conducted according to 2009 PRISMA guidelines [35].

### Search strategy

**Figure 1:**
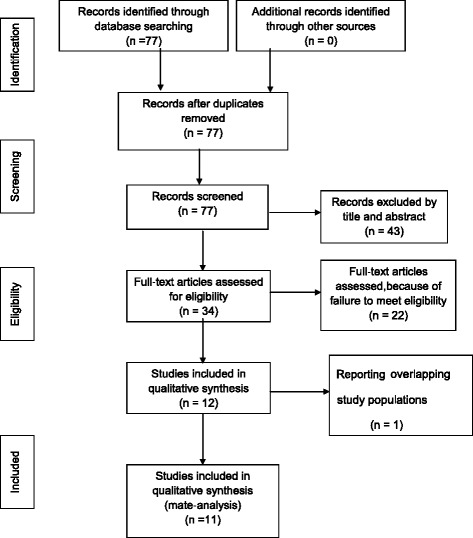
**Flow diagram of study selection according to the PRISMA statement.**

We performed a systematic research of available studies that assessed the association between *ERα* polymorphisms and OA. We carried out a comprehensive literature search for published studies in OVID MEDLINE, EMBASE, CBM, and PubMed databases, and Google Scholar. Primary key search terms included estrogen receptor, polymorphism, osteoarthritis, and OA. Index terms for OVID MEDLINE were: “estrogen receptor”, “polymorphism”, and “osteoarthritis” or “OA”. The last query was updated on 30 November 2014. There were no language or other limitations on the search. Reference lists in the retrieved articles or relevant reviews were also screened to identify other eligible studies. We also searched unpublished studies by contacting clinical experts and the Arthritis Foundation National Office. A flow diagram of our literature identification strategy is shown in Figure 1.

### Inclusion and exclusion criteria

Eligible studies were required to satisfy the following criteria: (1) the study was a cohort or a case–control study; (2) OA was diagnosed based on clinical criteria defined by the American College of Rheumatology; (3) the original study assessed the association between *ERα* polymorphisms (*Xba*I or *Pvu*II) and OA susceptibility; and (4) the study provided sufficient genetic frequency or sufficient data for extraction. If overlapping study populations were identified between studies, only the most complete one was included in the meta-analysis. Animal studies and literature reviews were excluded.

### Quality assessment of included studies

Study quality was independently assessed by two authors, based on the Newcastle–Ottawa scale (NOS) quality score systems [36]. The NOS contains eight items divided into three categories: selection, comparability, and outcome (for cohort studies) or exposure (for case–control studies). Quality scores ranged from 0 to 9. When there was disagreement on the quality scores between the two authors, discrepancies were resolved through discussion and consultation with a third author.

The quality of included studies was also assessed by the Hardy–Weinberg equilibrium (HWE) for the control genotype distribution. Studies consistent with HWE were defined as high-quality, while those inconsistent with HWE were defined as low-quality studies.

### Data extraction

The following data were extracted from each full-text study using a standardized data extraction form: the name of the first author, year of publication, country in which the study was performed, study design, number of cases and controls, gender, age, genotyping, OA site, OA definition, polymorphism, and numbers of cases and controls for each of the *Pvu*II (rs2234693), and *Xba*I (rs9340799) genotypes. When the information extracted from studies was inconsistent, disagreement was resolved through discussion and consultation with a third author until consensus was achieved on every item.

### Statistical analysis

STATA 12.0 and Review Manager 5.2 software were used for data analysis. The pooled odds ratio (OR) and its 95% confidence interval (95% CI) were calculated to assess the association between *ERα* polymorphisms and the risk of OA for the following contrasts: G vs. A, AG vs. AA + GG, GG vs. AG + AA, AA vs. AG + GG, C vs. T, CC vs. TT + CT, CT vs. CC + TT, and TT vs. CC + CT. Subgroup analysis based on ethnicity was also performed. The Chi-square test was used to determine if the identified study was consistent with HWE for the control genotype distribution. Heterogeneity between studies was evaluated with the *I*^2^ test and the *Q* statistic. We used the Cochrane system for heterogeneity grading: *I*^2^ 0–40%, might not be important; 30–60%, moderate heterogeneity; 50–90%, substantial heterogeneity; 75–100%, considerable heterogeneity. Heterogeneity was assessed to be significant when *I*^2^ > 30% or when *P* < 0.1 for *Q* statistics.

The pooled effects were estimated using the Der-Simonian and Laird method for random effects and the Mantel–Haenszel method for fixed effects [37]. If the studies were significantly heterogeneous, we used the random effects model. Otherwise, we used the fixed effects model to calculate the pooled OR and 95%CI. The random effects model assumes that different studies have substantial diversity and assesses both within-study sampling error and between-study variation [38]. The fixed effects model assumes that genetic factors have similar effects on OA susceptibility across all studies, and that observed variations between studies are caused by chance alone [39]. Sensitivity analyses were performed for the effect size omitting the trial for which data were imputed, and were used to evaluate the stability of the results. Publication bias was graphically represented by funnel plots and further evaluated with the Begg’s test and Egger’s test [40,41].

## Results

### Search results and studies included in the meta-analysis

Seventy-seven relevant studies were preliminarily identified in the database search, of which 11 [24-34] eventually satisfied the eligibility criteria for our meta-analysis. All included studies investigated the relationship between *ERα* polymorphisms and OA susceptibility. Of these, one study [33] contained data on three different OA sites and four different geographical locations, so these seven comparisons were treated independently. Therefore, a total of 17 separate comparisons were included in the present meta-analysis. Ten studies with a total of 8,502 participants (2,181 OA patients and 6,321 controls), which involved three European and seven Asian populations, evaluated the association between the *ERα Xba*I polymorphism and OA susceptibility, while 17 with 16,159 total participants (5,325 OA patients and 10,834 controls), involving 10 European and seven Asian populations, evaluated the association between the *ERα Pvu*II polymorphism and OA susceptibility. Study characteristics are summarized in Table 1.

**Table 1:** **Characteristics of the included studies**

**Study [Ref.]**	**Year**	**Country (City)**	**Study design**	**Genotyping**	**Numbers**		**Gender (M/F)**		**Age**		**Polymorphism (s)**	**Quality score**
					**OA**	**Control**	**OA**	**Control**	**OA**	**Control**		
Toshio Ushiyama et al. [24]	1998	Japan	Case–control	PCR	65	318	0/65	0/318	68.5 (49–86)	49-86	XbaI, PvuII	7 (2/2/3)
John Loughlin et al. [29]	2000	UK (Oxford)	Case–control	PCR	371	369	155/216	221/148	73 (56–90)	73 (59–89)	XbaI, PvuII	8 (3/2/3)
Barton L. Wise et al. [30]	2009	USA	Cohort	PCR	307	214	258/263		61 ± 9		XbaI	8 (4/2/2)
Barton L. Wise et al. [30]	2009	USA	Cohort	PCR	304	211	253/262		61 ± 9		PvuII	
V. M. Borgonio-Cuadra et al. [32]	2012	Mexico	Case–control	PCR	115	117	23/92	20/97	57.4 ± 9.2	51.8 ± 8.9	XbaI, PvuII	9 (4/2/3)
J. A. Riancho et al. [33]	2010	Spain (Santander)	Case–control	PCR	272	802	95/177	285/517	72 ± 7	71 ± 10	PvuII	8 (3/2/3)
J. A. Riancho et al. [33]	2010	Spain (Santiago)	Case–control	PCR	254	473	47/207	295/178	68 ± 6	68 ± 9	PvuII	
J. A. Riancho et al. [33]	2010	UK (Oxford)	Case–control	PCR	445	862	176/269	471/391	64 ± 5	69 ± 7	PvuII	
J. A. Riancho et al. [33]	2010	Spain (Santander)	Case–control	PCR	359	802	180/179	285/517	71 ± 7	71 ± 10	PvuII	
J. A. Riancho et al. [33]	2010	Spain (Coruña)	Case–control	PCR	252	244	90/162	97/147	67 ± 14	65 ± 13	PvuII	
J. A. Riancho et al. [33]	2010	Spain (Santiago)	Case–control	PCR	287	473	110/177	295/178	68 ± 5	68 ± 9	PvuII	
J. A. Riancho et al. [33]	2010	UK (Oxford)	Case–control	PCR	1278	862	503/775	471/391	65 ± 6	69 ± 7	PvuII	
K. Lian M.D. et al. [31]	2007	USA	Cohort	PCR	569	4134	0/569	0/4134	79.6 ± 5.0	78.4 ± 4.6	XbaI, PvuII	8 (4/2/2)
Sheng-Yu Jin et al. [25]	2004	Korea	Case–control	PCR	151	397	53/98	190/207	58.8 ± 9.6	/	XbaI, PvuII	8 (3/2/3)
Zhi Tian et al. [28]	2009	China	Case–control	PCR	38	40	0/38	0/40	59.2 ± 3.2	58.5 ± 8.6	XbaI, PvuII	7 (2/2/3)
Jiexiang Yang et al. [34]	2009	China	Case–control	PCR	41	40	31/50		54.6 (28–82)		XbaI, PvuII	6 (2/1/3)
Yan Xue et al. [27]	2004	China	Case–control	PCR	55	176	0/55	0/176	58.7 ± 2.4	60 ± 10	XbaI, PvuII	7 (3/1/3)
Xiaoyu Dai et al. [26]	2014	China	Case–control	PCR	469	522	113/356	398/124	57.3 ± 10.9	56.4 ± 9.8	XbaI, PvuII	7 (3/1/3)

### Allele and genotype counts

Allelic counts of the *ERα Xba*I polymorphism were evaluated for G and A alleles. In general, the frequency of the A allele was higher in OA cases than in controls. Genotype counts of the *ERα Xba*I polymorphism were evaluated for GG, AG, and AA genotypes, and the frequency of the AA genotype was higher in OA cases than in the control group in all but one study [24]. The frequency of the AG genotype was lower in OA cases than in the control group in all but the same study [24]. There was no obvious difference in the frequency of the GG genotype between OA cases and controls. Allele and genotype counts for the *ERα Xba*I polymorphism in cases and controls are shown in Table 2.

**Table 2:** **Genotype and allele counts for the** ***ERα Xba*** **I polymorphism in the included studies**

**Group**	**Study**	**Country**	**OA site**	**X (G)**		**x (A)**		**xx (AA)**		**Xx (AG)**		**XX (GG)**	
				**OA**	**Control**	**OA**	**Control**	**OA**	**Control**	**OA**	**Control**	**OA**	**Control**
European	Barton L. Wise et al.	USA	Hand	202	159	412	269	148	85	116	99	43	30
	John Loughlin et al.	UK	Hip,Knee	256	251	486	487	164	161	158	165	49	43
	K. Lian M.D. et al.	USA	Hip	374	2914	764	5332	257	1700	250	1932	62	491
European Total				832	3324	1662	6088	569	1946	524	2196	154	564
Asian	Sheng-Yu Jin et al.	Korea	Knee	57	156	245	638	98	256	49	126	4	15
	Toshio Ushiyama et al.	Japan	Hand	30	116	100	520	36	211	28	98	1	9
	V. M. Borgonio-Cuadra et al.	Mexico	Knee	49	63	181	171	70	62	41	47	4	8
	Zhi Tian et al.	China	Knee	24	47	52	33	18	6	16	21	4	13
	Jiexiang Yang et al.	China	Knee	15	19	67	61	28	24	11	13	2	3
	Yan Xue et al.	China	Knee	44	200	66	162	21	40	24	82	10	54
	Xiaoyu Dai et al.	China	Knee	210	193	728	851	288	348	152	155	29	19
Asian Total				429	794	1439	2436	559	947	321	542	54	121
Total				1261	4118	3101	8524	1128	2893	845	2738	208	685

Allelic counts of the *ERα Pvu*II polymorphism were evaluated for C and T alleles. In general the T allele frequency was higher in OA cases than in the control group. Genotype counts of the *ERα Pvu*II polymorphisms were evaluated for TT, CT, and CC genotypes, and the TT genotype frequency was generally higher in OA cases than in controls. The CC genotype frequency was generally lower in OA cases than controls, although there was no obvious difference in the frequency of the CT genotype between the two groups. Allele and genotype counts for the *ERα Pvu*II polymorphism in cases and controls are shown in Table 3.

**Table 3:** **Genotype and allele counts for the** ***ERα Pvu*** **II polymorphism in the included studies**

**Group**	**Study**	**Coutry (City)**	**OA site**	**P (C)**		**p (T)**		**pp (TT)**		**Pp (CT)**		**PP (CC)**	
				**OA**	**Control**	**OA**	**Control**	**OA**	**Control**	**OA**	**Control**	**OA**	**Control**
European	Barton L. Wise et al.	USA	Hand	261	192	347	230	101	65	145	100	58	46
	J. A. Riancho et al.	Spain (Coruña)	Hip	213	217	291	271	89	76	113	119	50	49
	J. A. Riancho et al.	UK (Oxford)	Hip	1109	776	1447	948	426	253	595	442	257	167
	J. A. Riancho et al.	UK (Oxford)	Knee	399	776	491	948	123	253	245	442	77	167
	J. A. Riancho et al.	Spain (Santander)	Hip	334	752	384	852	105	229	174	394	80	179
	J. A. Riancho et al.	Spain (Santander)	Knee	246	752	298	852	79	229	140	394	53	179
	J. A. Riancho et al.	Spain (Santiago)	Knee	235	377	273	569	65	176	143	217	46	80
	J. A. Riancho et al.	Spain (Santiago)	Hip	239	377	335	569	99	176	137	217	51	80
	John Loughlin et al.	UK	Hip,Knee	331	331	411	407	114	110	183	187	74	72
	K. Lian M.D et al.	USA	Hip	481	3835	653	4391	188	1162	277	2067	102	884
European Total				3848	8385	4930	10037	1389	2729	2152	4579	848	1903
Asian	Sheng-Yu Jin et al.	Korea	Knee	112	307	190	487	61	152	68	183	22	62
	Toshio Ushiyama et al.	Japan	Hand	57	260	73	376	19	115	35	146	11	57
	V. M. Borgonio-Cuadra et al.	Mexico	Knee	77	82	153	152	52	51	49	50	14	16
	Zhi Tian et al.	China	Knee	29	34	47	46	16	15	15	16	7	9
	Jiexiang Yang et al.	China	Knee	37	33	45	47	14	12	17	23	10	5
	Yan Xue et al.	China	Knee	53	151	57	201	17	57	23	87	15	32
	Xiaoyu Dai et al.	China	Knee	387	390	551	638	167	198	217	242	85	74
Asian Total				752	1257	1116	1947	346	600	424	747	164	255
Total				4600	9642	6046	11984	1735	3329	2576	5326	1012	2158

### Quality assessment of included studies

All 11 studies had a satisfactory NOS quality score as shown in Table 1. The distribution of genotypes in the controls was in accordance with HWE (*P* > 0.05) in all studies, so all were classed as high-quality.

### Meta-analysis findings

**Figure 2:**
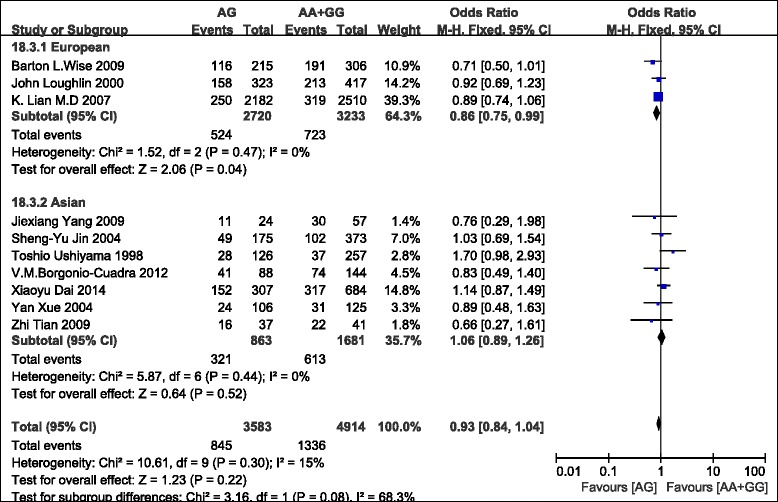
**Meta-analysis of the association between the** ***ERα Xba*** **I polymorphism and OA (AG vs. AA + GG).**

A summary of the meta-analysis findings are shown in Table 4. The *ERα Xba*I polymorphism was shown not to be associated with OA risk in all populations (G vs. A: OR = 0.87, 95% CI = 0.73–1.04, *P* = 0.13; AA vs. AG + GG: OR = 1.16, 95% CI =0.94–1.44, P = 0.17; AG vs. AA + GG: OR = 0.93, 95% CI =0.84–1.04, *P* = 0.22; GG vs. AG + AA: OR = 0.88, 95% CI =0.67–1.17, *P* = 0.38). However, subgroup analysis by ethnicity showed that the AA and AG genotypes of the *ERα Xba*I polymorphism were associated with OA risk among Europeans (AA vs. AG + GG: OR = 1.17, 95% CI = 1.02–1.34, *P* = 0.03; AG vs. AA + GG: OR = 0.86, 95% CI = 0.75–0.99, *P* = 0.04), but not among Asian populations (Figure 2).

**Table 4:** **Meta-analysis of** ***ERα Xba*** **I and** ***Pvu*** **II polymorphisms and OA susceptibility**

**Polymorphism comparison**	**Population OA site**	**No. of studies**	**Test of association**			**Test of heterogeneity**				**Test of publication bias**			
										**Begg's test**		**Egger's test**	
			**OR**	**95% ** ***CI***	***p*** **-value**	**Model**	**Q test**	***p*** **-value**	***I*** ^**2**^	**Z test**	***p-value***	**T test**	***p-value***
**XbaI** (G vs. A)	Overall	10	0.87	0.73—1.04	0.13	Random	29.71	0.0005	70%	−0.80	0.42	−1.29	0.23
	European	3	0.91	0.82—1.01	0.08	Fixed	1.67	0.43	0%				
	Asian	7	0.80	0.57—1.13	0.21	Random	27.74	0.0001	78%				
AA vs. AG + GG	Overall	10	1.16	0.94—1.44	0.17	Random	25.55	0.002	65%	1.16	0.25	0.94	0.38
	European	3	1.17	1.02—1.34	0.03	Fixed	1.91	0.39	0%				
	Asian	7	1.22	0.84—1.79	0.30	Random	21.69	0.001	72%				
AG vs. AA + GG	Overall	10	0.93	0.84—1.04	0.22	Fixed	10.61	0.30	15%	0.09	0.93	0.47	0.65
	European	3	0.86	0.75—0.99	0.04	Fixed	1.52	0.47	0%				
	Asian	7	1.06	0.89—1.26	0.52	Fixed	5.87	0.44	0%				
GG vs. AG + AA	Overall	10	0.88	0.67—1.17	0.38	Random	14.23	0.11	37%	−0.80	0.42	1.89	0.10
	European	3	0.97	0.79—1.20	0.81	Fixed	0.85	0.65	0%				
	Asian	7	0.65	0.36—1.19	0.17	Random	12.58	0.05	52%				
**PvuII** (C vs. T)	Overall	17	0.98	0.93—1.03	0.37	Fixed	19.58	0.24	18%	0.66	0.51	0.87	0.40
	European	10	0.97	0.90—1.04	0.14	Random	13.61	0.14	34%				
	Asian	7	1.07	0.95—1.21	0.25	Fixed	3.19	0.78	0%				
CC vs. TT + CT	Overall	17	0.97	0.89—1.06	0.55	Fixed	13.09	0.67	0%	0.08	0.93	0.26	0.80
	European	10	0.94	0.85—1.04	0.21	Fixed	5.05	0.83	0%				
	Asian	7	1.17	0.94—1.47	0.16	Fixed	4.94	0.55	0%				
CT vs. CC + TT	Overall	17	0.99	0.92—1.06	0.75	Fixed	19.85	0.23	19%	0.41	0.68	0.73	0.48
	European	10	1.01	0.91—1.13	0.82	Random	15.43	0.08	42%				
	Asian	7	0.96	0.81—1.14	0.64	Fixed	4.29	0.64	0%				
TT vs. CC + CT	Overall	17	1.01	0.92—1.12	0.79	Random	23.83	0.09	33%	−0.25	0.81	−1.15	0.27
	European	10	1.02	0.90—1.17	0.74	Random	20.33	0.02	56%				
	Asian	7	0.95	0.80—1.13	0.57	Fixed	2.35	0.88	0%

**Figure 3:**
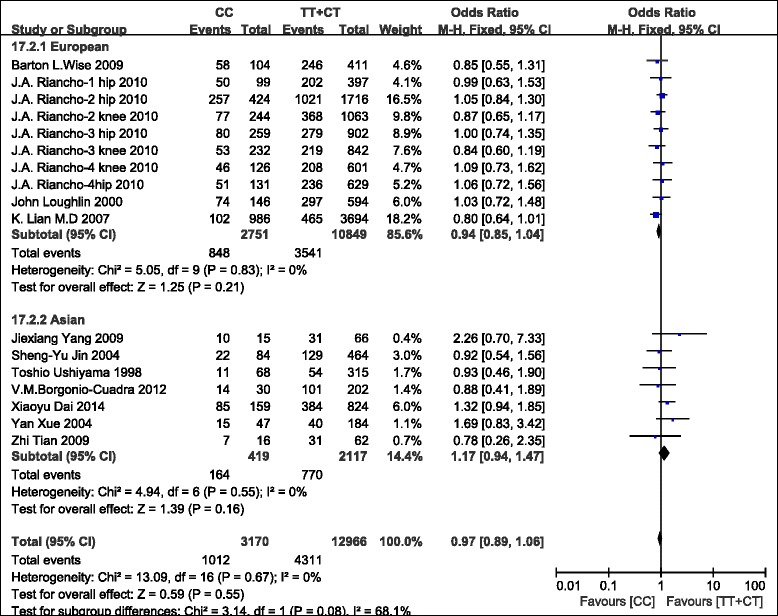
**Meta-analysis of the association between the** ***ERα Pvu*** **II polymorphism and OA (CC vs. TT + CT).**

There was no significant association between the *ERα Pvu*II polymorphism and susceptibility to OA in all populations (C vs. T, OR = 0.98, 95% CI = 0.93–1.03, *P* = 0.37; CC vs. TT + CT, OR = 0.97, 95% CI = 0.89–1.06, *P* = 0.55; CT vs. CC + TT, OR = 0.99, 95% CI = 0.92–1.06, *P* = 0.75; TT vs. CC + CT, OR = 1.01, 95% CI = 0.92–1.12, *P* = 0.79). In the subgroup analysis based on ethnicity, no significant association was found for the *ERα Pvu*II polymorphism in either European or Asian populations (Figure 3).

### Sensitivity analysis and publication bias

As shown in Table 4, heterogeneity was observed among studies in all populations and also in subgroup analyses. To explore the sources of heterogeneity across studies we performed a sensitivity analysis, which revealed that none of the studies significantly affected the pooled ORs and CIs. Sequential removal of each study had little effect on the pooled ORs.

**Figure 4:**
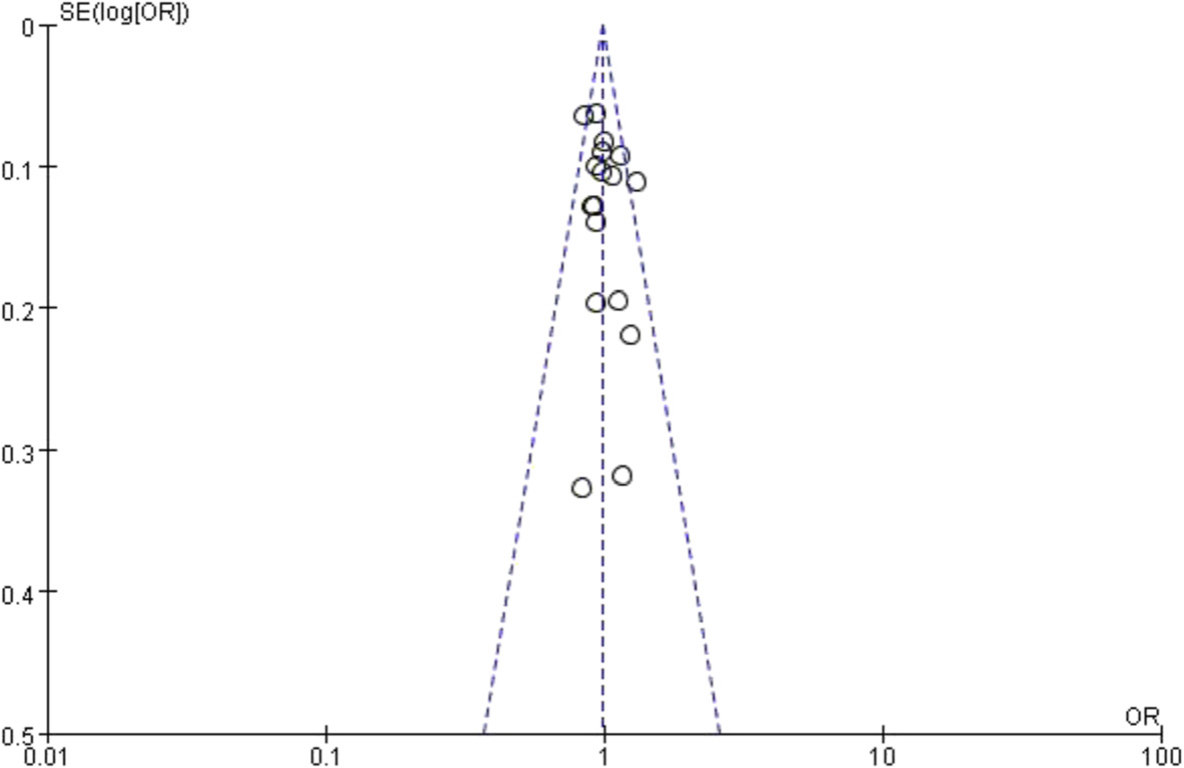
**Funnel plot of the meta-analysis of the ERα PvuII polymorphism with susceptibility to OA (C vs. T).**

The funnel plot revealed no obvious publication bias (Figure 4), and this was confirmed by Begg’s test and Egger’s test.

## Discussion

Although the pathogenesis of OA is considered to be the result of many factors, genetics are thought to be one of the most important determinants [42]. Despite the fact that *ERα* is one of the most studied genes in OA [43], to the best of our knowledge this is the first meta-analysis of the relationship between *ERα* polymorphisms *Xba*I and *Pvu*II and OA risk.

Our meta-analysis included 11 published studies (with 17 comparisons) of 16,159 participants (5,325 OA patients and 10,834 controls). Ten studies with a total of 8,502 participants evaluated the association between the *ERα Xba*I polymorphism and OA susceptibility, and our meta-analysis suggested that it was significantly associated with OA in European but not Asian populations. The pooled OR for homozygote AA carriers showed that they were associated with a 17% increased risk for OA compared with AG and GG carriers, and that European AG carriers had a decreased OA risk. The heterogeneity of genetic effects between European and Asian populations suggests the existence of gene–environment or gene–gene interactions. No heterogeneity was detected in European populations with respect to the *ERα Xba*I polymorphism and OA, suggesting that the genetic effect of this polymorphism is stronger in European than Asian populations.

Seventeen studies with a total of 16,159 participants evaluated the association between the *ERα Pvu*II polymorphism and OA susceptibility. Our meta-analysis suggested that there was no association between the polymorphism and susceptibility to OA in any population. The same result was obtained for the subgroup analysis based on ethnicity.

Gender differences are known to affect the development of OA; for example, the prevalence of knee OA is greater in women than men [15]. Only two of the studies included in our meta-analysis were stratified according to participant gender [25,32], and both reported no significant differences in the *ERα* polymorphisms between OA patients and controls of the same sex. However, because of the small number of this type of study and the limited raw data based on gender differences in genotype distributions and allele frequencies, we were unable to perform a subgroup analysis according to gender.

Several limitations should be taken into consideration in the current meta-analysis. First, it was based on unadjusted OR estimates because not all studies presented adjusted ORs, or the ORs were not adjusted by the same potential confounders, such as age and gender. This lack of information could have caused serious confounding bias. Second, OA is influenced by both genetic and environmental risk factors such as obesity, injury, occupational activities, and meniscectomy. However, the studies included in the meta-analysis did not control for these environmental risk factors. Third, some studies included individuals with OA in different sites, but we were unable to perform subgroup analysis of this within the same ethnic population because of the limited available data. For instance, hand OA is known to be more influenced by genetic and hormonal influences than other types of OA, but the relationship between the *ERα Xba*I polymorphism and hand OA was only reviewed in one study of Europeans [30] and one of Asians [24]. Other studies of the *ERα Xba*I polymorphism and OA susceptibility in Europeans focused on three different OA sites. Finally, although our current findings suggest that the *ERα Xba*I polymorphism is associated with OA in Europeans, it was not possible to determine whether this polymorphism is in linkage disequilibrium with any other potentially functional polymorphisms. However, our meta-analysis also had some advantages, including a satisfactory quality of all 11 included studies, and a well-designed method.

## Conclusions

The present results suggest that there may be a weak relationship between the *ERα Xba*I polymorphism and OA in European but not Asian populations, while the *ERα Pvu*II polymorphism did not appear to be associated with OA in either Europeans or Asians. Because the studies included in the meta-analysis reviewed the relationship between the *ERα Xba*I polymorphism and OA susceptibility at three different sites in Europeans, large well-designed studies are necessary to confirm our findings in more homogeneous populations.
